# IDH1 R132H and TP53 R248Q Mutations Modulate Glioma Cell Migration and Adhesion on Different ECM Components

**DOI:** 10.3390/ijms252212178

**Published:** 2024-11-13

**Authors:** Mikhail E. Shmelev, Andrei A. Pilnik, Nikita A. Shved, Alina O. Penkova, Valeriia S. Gulaia, Vadim V. Kumeiko

**Affiliations:** 1 School of Medicine and Life Sciences, Far Eastern Federal University, Vladivostok 690922, Russia; 2 A.V. Zhirmunsky National Scientific Center of Marine Biology Far Eastern Branch of Russian Academy of Sciences, Vladivostok 690041, Russia

**Keywords:** brain matrix, atomic force microscopy, laminin, chondroitin sulfate, hyaluronic acid, collagen IV, collagen I, Matrigel

## Abstract

Mutations in IDH1 and TP53 have a significant impact on glioma prognosis and progression; however, their roles in tumor cell invasion in terms of interactions with particular components of the extracellular matrix (ECM) are still unclear. Using gene editing protocol based on CRISPR-Cas 9 with cytidine deaminase, we introduced point mutations into U87MG glioblastoma cells to establish modified cell lines with heterozygous IDH1 R132H, homozygous TP53 R248Q and heterozygous IDH1 R132H, homozygous TP53 R248Q genotypes. A comparative study of cell migration on major ECM components was carried out by high-content microscopy. IDH1 R132H mutation introduced to U87MG glioblastoma cells was shown to decrease the migration speed on Matrigel and collagen IV substrates compared to the wild-type. This data were supported by cell adhesion quantification via the lateral shift assay performed by atomic force microscopy (AFM). TP53 R248Q mutation increased cell adhesion to various substrates and significantly promoted cell migration on hyaluronic acid and chondroitin sulfate but did not change the migration rates on laminin and collagens IV and I. A double-mutant genotype produced by consequently introducing IDH1 R132H and TP53 R248Q to parental glioblastoma cells was characterized by the highest migration among all the cell lines, with particularly faster motility on chondroitin sulfate. These findings underscore the complex interactions between glioma cells, with the most important driver mutations and specific ECM components regulating cancer cell migration, offering valuable insights for potential therapeutic targets in glioma treatment.

## 1. Introduction

Glioblastoma (GB) is one of the most aggressive malignancies and also the most common malignant primary tumors of the central nervous system (CNS), with a median five-year survival rate of 5.4%, according to the World Health Organization (WHO) [1]. In fact, 5-year survival remains poor, with no apparent improvement over the past decade [2]; in this regard, the problem of GB investigation has become pivotal and is in high demand. Mutations in IDH1 and TP53 are crucial for cancer diagnostics and prognosis. IDH1 R132H mutation is associated with better clinical outcomes, and this mutation is a main diagnostic criterion to differentiate low-grade diffuse gliomas and to administer target therapy. Mutations in TP53 are important for the diagnostics of several glioma subtypes: IDH-mutant astrocytoma, H3 K27-altered diffuse midline glioma, H3 G34-mutant diffuse hemispheric glioma and SHH-activated medulloblastoma [3]. According to the Tumor Cancer Genome Atlas Genomic Data Commons database (TCGA GDC database), the most common point mutations in TP53 gene are R273C, R175H and R248Q. The mutation with the greatest negative effect on survival is R248Q (https://portal.gdc.cancer.gov/, accessed on 12 November 2024). Thus, the TP53 R248Q sequence variant is of clinical interest for diagnosis and survival prognosis. The GB is characterized by relatively high levels of invasion compared to other types of cancer cells. Tumor cell migration is directly dependent on interaction with the extracellular matrix (ECM) [4]. Moreover, transforming the ECM occurs during GB development, which enhances the cancer progression [5].

The GB migratory capacity is determined by myriad factors in the tumor microenvironment (TME), including the ECM, blood vessels and a set of different soluble factors and adjacent cells [6,7].

The brain tissue ECM is a unique structure, containing proteins and carbohydrate components. On the one hand, the most important and best-studied structural proteins of the ECM are collagens, fibronectins and laminins [7]. The frequent type of collagen that meets in the brain ECM is type IV, which creates a network by association with non-collagenous (NC1) domains [8]. Laminins contribute to glioma cell migration and invasion. Furthermore, laminin is located in the tumor parenchyma and tumor invasion front and is one of the main blood vessels components [9]. Fibronectin is also found around blood vessels and provides glioma migration from the perivascular niche into the brain parenchyma [10].

On the other hand, no less important and well-studied ECM structures are carbohydrate components that could be classified into glycosaminoglycans (GAGs) and proteoglycans, such as chondroitin sulfates (CS), heparin sulfate and keratan sulfate [11]. GAGs do not contain a protein core like other proteoglycans, and they play a crucial role in the cell–matrix signaling pathways [9,12,13]. The major GAG presented in the brain ECM is hyaluronic acid (HA) [9]. Proteoglycans act as connectors between hyaluronic acid, tenascin-R and link proteins, together forming perineuronal networks (PNNs) [14]. Thus, the ECM is a complicated organized structure that has properties, such as the quantity and quality content, rigidity and mutual location, that influence cell behavior and transformation.

The migration activity of GB cells is controlled by the unique structure of the ECM. In oncogenesis, GB cells transform the surrounding matrix enhancing the cell movement capacity. In the modified ECM, an increase in components such as hyaluronic acid, collagens, fibronectin, laminin and tenascin-R is observed [15].

Fibronectin and hyaluronic acid enhanced the migratory capacity and invasion of GB cells by direct interactions of integrin–fibronectin and CD44–hyaluronic acid, respectively [5,16]. Moreover, the ECM rigidity increases due to the upregulated synthesis of these ECM components, which also affects the migratory activity [17]. However, the majority of tumor cells migrating along blood vessels is accompanied by ECM remodeling, which could promote glioma–stroma interaction and tumor cell invasion into the surrounding brain [13]. 

Cell migration activity is determined not only by the ECM but also by the genetic profile. A typical glioblastoma contains more than 60 genetic alterations [18]. Therefore, IDH1-mutant gliomas demonstrate a high level of oncometabolite D-2-hydroxyglutarate, which causes an increase in migration activity through the PI3K/AKT/mTOR pathway and morphology changes [19,20]. However, some papers demonstrate inhibited motility of glioma cells with the IDH1 R132H mutation compared to IDH1 wild-type cells [21,22]. 

These data suggest that mutant IDH1 may promote glioma growth through transcriptional and non-transcriptional mechanisms that are independent of its epigenetic effects [23]. In contrast, the combination of IDH1 mutation and 1p/19q codeletion attributed to oligodendrogliomas decreases the migration activity of GB cells [24]. 

The TP53 mutation upregulates the migratory capacity through the regulation of Rho signaling, which controls actin cytoskeletal organization and prevents filopodia formation and migration. However, the loss of function of TP53 enhances the activities of RhoA and Rac, which promote cell adhesion and motility by activation of PI3K/AKT/mTOR signaling [18]. Interestingly, patient-derived early-passage glioma cell cultures with mutations in both IDH1 and TP53 are characterized with low proliferation and committed to a differentiation phenotype, which promotes high migratory ability [25]. 

We aimed to create a specific mutational landscape that mimics certain processes observed in some important clinical cases where IDH1R132H or TP53 R248Q play important roles. The combination of IDH1 R132H with TP53 R248Q allows us to investigate the effects of a double-mutant genotype on cellular behavior, specifically migration. The interplay between these mutations provides insights into their synergistic or antagonistic effects, which may not be captured by examining the mutations in isolation. The existing literary sources provide insufficient data regarding the correlation between the IDH1 and TP53 variants and migration abilities of glioma cells. The intricate relationship between driver mutations and glioma cell migratory capacity necessitates further investigation. Using gene editing protocol based on CRISPR-Cas 9 with cytidine deaminase, we established genetically modified U87MG glioblastoma cell lines with the heterozygous IDH1 R132H genotype (IDH1het), homozygous TP53 R248Q genotype (TP53homo) and combined heterozygous IDH1 R132H, homozygous TP53 R248Q genotype (IDH1het-TP53homo) to promote a comprehensive analysis of cell migration and adhesion on major ECM components, aiming to elucidate IDH1 R132H and TP53 R248Q’s impact on glioma cell migration.

## 2. Results

We conducted a series of experiments aimed at gene editing of the glioblastoma U87MG cell line to introduce genotypes with mutations IDH1 R132H, TP53 R248Q and their combination, coating culture substrates with major ECM components, assessing cell migration activity and evaluating the adhesive properties of cells to the extracellular matrix. The cellular models employed in this research enable the assessment of migration rate of glioma cells with various mutational profiles that are commonly encountered in clinical practice. High-content microscopy with individual cell tracking was applied for high-throughput cell population analysis for evaluating their migration rates. While cultivating on the specific substrate, each sample included more than 5000 cells for the measurement.

The methodology for direct measurements of cell adhesion to the extracellular matrix was implemented using atomic force microscopy (AFM) with a lateral shift assay. This structured approach allows for a comprehensive understanding of the cellular behaviors influenced by specific genetic alterations, contributing to the broader knowledge of cell–matrix interactions in the context of tumor biology.

### 2.1. Glioma Cell Lines Propagation and Molecular Profiling

**Figure 1:**
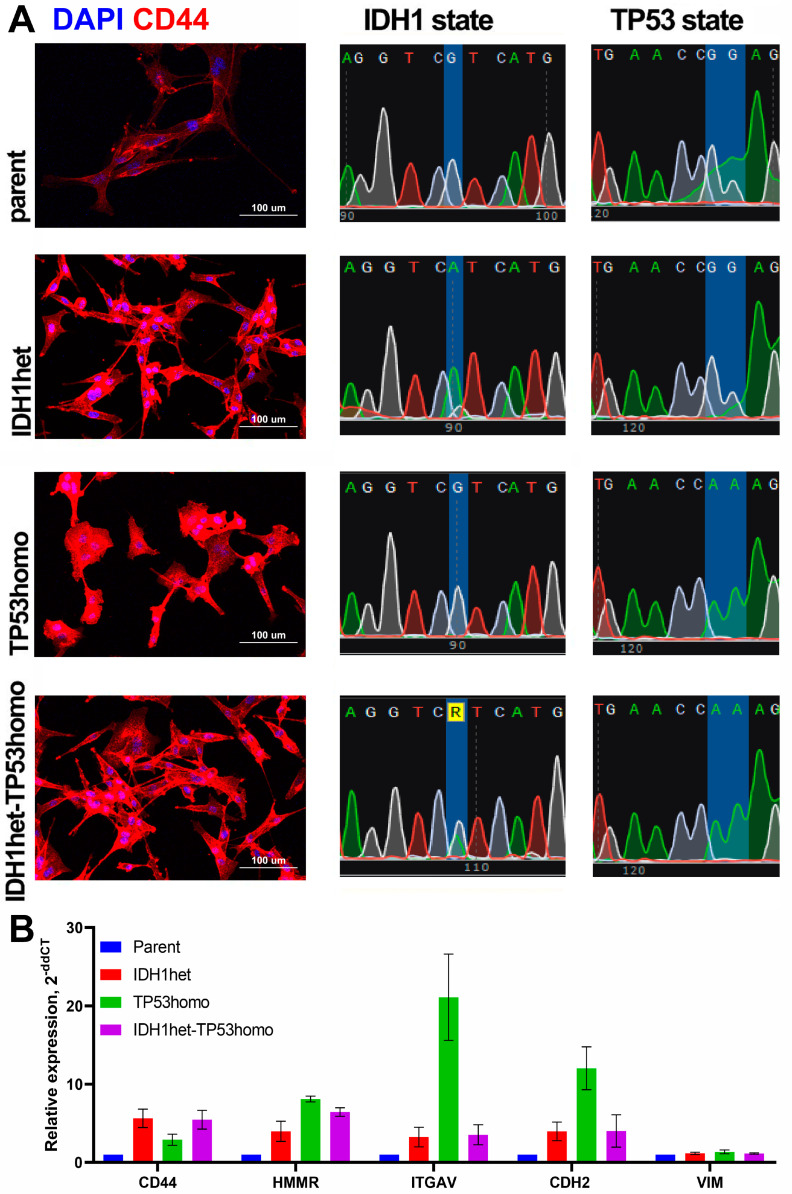
Molecular profiling of established glioma cell lines. (**A**) Representative images of each cell line stained for CD44 (red) and DAPI (blue) staining. Sequencing chromatograms for each cell culture. (**B**) Gene expression provided via qPCR assay.

We evaluated the impact of driver mutations in the IDH1 and TP53 genes on the interaction between cells and different components of the ECM. Cell lines characterized by the heterozygous IDH1 R132H genotype (IDH1het), heterozygous TP53 R248Q genotype (TP53homo) and combined heterozygous IDH1 R132H, homozygous TP53 R248Q genotype (IDH1het-TP53homo) were established from the U87MG initial cell line (U87MG Parent) using CRISPR-Cas9 with cytidine deaminases. All cell lines were proven for the genotypes established and CD44 expression (Figure 1A). A high expression of CD44 was found in the double-mutant cell culture IDH1het-TP53homo that inspired us to estimate differential cell migration on various ECM substrates, including hyaluronic acid, which is responsible for an interaction with CD44 and the mesenchymal-like cell phenotype.

To assess the influence of IDH1 and TP53 mutations on the molecular markers of cell migration, we conducted a qPCR gene expression assay (Figure 1B). The IDH1 R132H mutant cell lines exhibited an elevated expression of CD44. The TP53 R248Q mutant cell lines displayed a significantly increased expression of HMMR. The TP53 homozygous cell line was characterized by markedly higher levels of ITGAV and CDH2 expression. Conversely, the double-mutant IDH1 heterozygous-TP53 homozygous cell line demonstrated a reduced expression of ITGAV and CDH2, akin to the IDH1 heterozygous cell line. All cell lines exhibited comparable levels of vimentin expression. More intriguingly, double-mutant IDH1het-TP53homo is characterized by a high expression of CD44 and HMMR, both responsible for the molecular recognition of HA and CS.

### 2.2. Surface Coating for the Cell Culture

**Figure 2:**
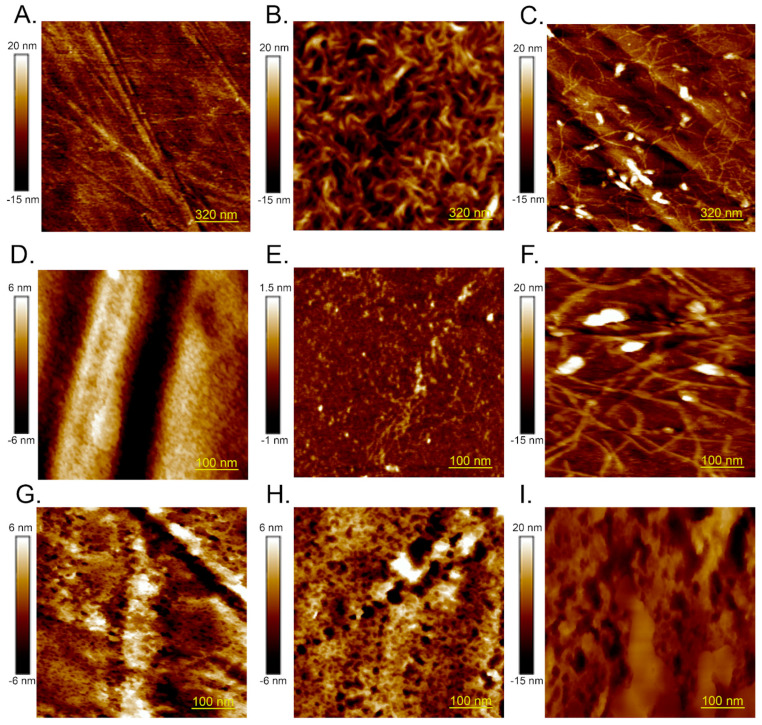
Topographical AFM images of ECM materials on cell culture-treated polystyrene: (**A**) polystyrene without coating; (**B**) collagen I; (**C**) laminin; (**D**) polystyrene; (**E**) collagen IV; (**F**) laminin; (**G**) chondroitin 6-sulfate; (**H**) hyaluronic acid; (**I**) Matrigel.

The cell culture coatings were evaluated using atomic force microscopy (AFM), revealing distinct morphological differences among the various ECM components. The control sample, consisting of an uncoated plastic dish, exhibited long, thin, straight polystyrene structures that facilitated cell adhesion (Figure 2A,D). In contrast, the surfaces coated with collagen I, collagen IV and laminin displayed long, curved protein fibers (Figure 2B,C,E,F).

Specifically, collagen I formed a network composed of thick fibers (Figure 2B), while laminin was characterized by long fibers with a terminal coiled-coil domain (Figure 2C,F). Collagen IV also exhibited a thin network structure, with the fibers displaying characteristic globular NC1 domains (Figure 2E). Polysaccharide-coated matrices, including HA and CS, and composite coating Matrigel presented sponge-like textures (Figure 2G–I).

### 2.3. TP53 R248Q Mutation Increases Cell Migration on Polysaccharide Substrates, and IDH1 R132H Reduces Cell Motility on Collagen IV and Matrigel

**Figure 3:**
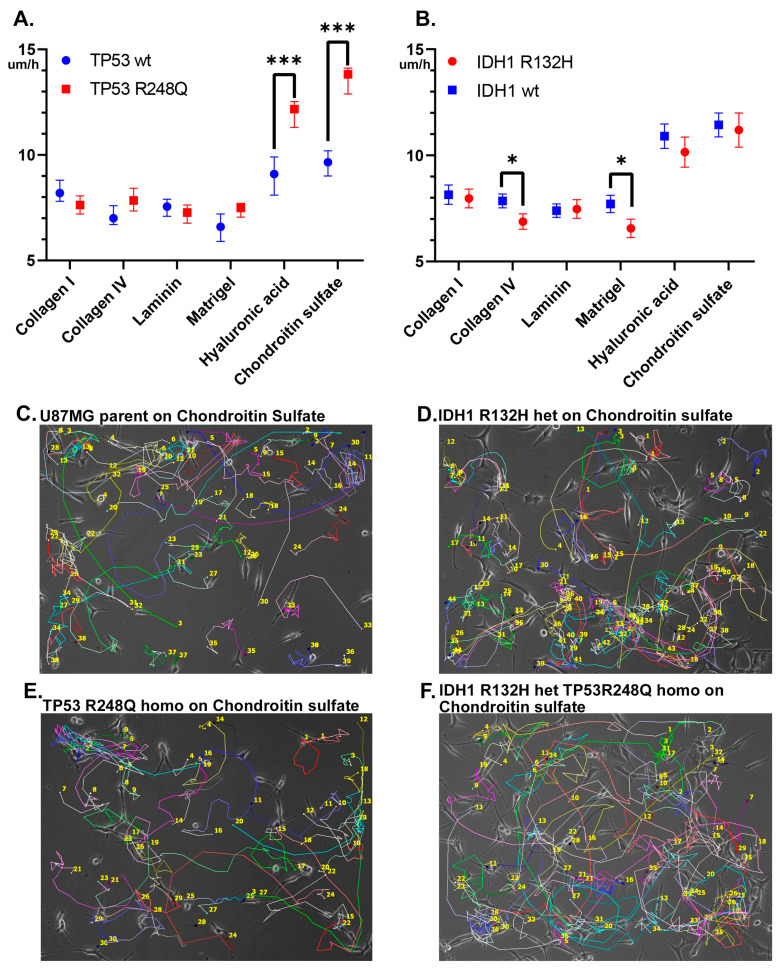
Mutant cell migration on various surfaces. Asterisks indicate level of statistical significance: *** *p* ≤ 0.001, * *p* ≤ 0.05 (**A**) Comparison of cell migration depending on the presence of the TP53 R248Q mutation and matrix material. (**B**) Comparison of cell migration depending on the presence of the IDH1 R132H mutation and matrix material. (**C**) Cell track of the U87MG parent cell line on chondroitin sulfate. (**D**) Cell track of the U87MG IDH1 R132H het cell line on chondroitin sulfate. (**E**) Cell track of the U87MG TP53 R248Q homo cell line on chondroitin sulfate. (**F**) Cell track of the U87MG IDH1 R132H het TP53 R248Q homo cell line on chondroitin sulfate.

It has been established that the presence of the TP53 R248Q mutation significantly influences the migratory activity of cells when cultured on polysaccharide matrices, namely, hyaluronic acid and chondroitin sulfate. In cultures grown on these matrices, the median migratory activity for cell lines grouped as one sample bearing the TP53 R248Q mutation (TP53homo and IDH1het-TP53homo) was 12.17 µm/h (95% CI of the median 11.3–12.53 µm/h) and 13.82 µm/h (95% CI of the median 12.89–14.11 µm/h) for hyaluronic acid and chondroitin sulfate, respectively. In contrast, the median migratory activity for the combined sample without the TP53 R248Q mutation (U87 MG parent and IDH1het) was 9.1 µm/h (95% CI of the median 8.1–9.9 µm/h) and 9.65 µm/h (95% CI of the median 9–10.2 µm/h) for hyaluronic acid and chondroitin sulfate, respectively (Figure 3A). When comparing migration of the cell lines with the TP53 R248Q mutation (Figure 3E,F) to those with wild-type TP53 on HA (Figure 3C,D), the significance level was *p* = 0.0001; for the same samples on CS, it was *p* = 0.0002 (Figure 3A).

No significant effect of IDH1 R132H substitution on cell migration found on the polysaccharide matrices (*p* > 0.05) (Figure 3B). However, the IDH1 R132H mutation is associated with significantly (*p* < 0.05) lower cell migration on collagen IV and Matrigel (Figure 3B). In cultures grown on these matrices, the median migratory activity for those with the IDH1 R132H (IDH1 het and TDH1het-TP53homo) mutation was 6.25 µm/h (95% CI of the median 5.8–7.13 µm/h) and 7 µm/h (95% CI of the median 6.0–7.56 µm/h) for Matrigel acid and collagen IV, respectively. In contrast, the median migratory activity for cultures without the IDH1 R132H mutation (U87MG parent and TP53homo) was 7.41 µm/h (95% CI of the median 7.03–7.99 µm/h) and 7.85 µm/h (95% CI of the median 7.2–8.3 µm/h) for Matrigel acid and collagen IV, respectively (Figure 3B).

### 2.4. IDH1 R132H Mutation Reduces Cell Migration Ability on Matrigel and Collagen IV, but Its Co-Occurence with TP53 R248Q Recovers High Cell Migration

**Figure 4:**
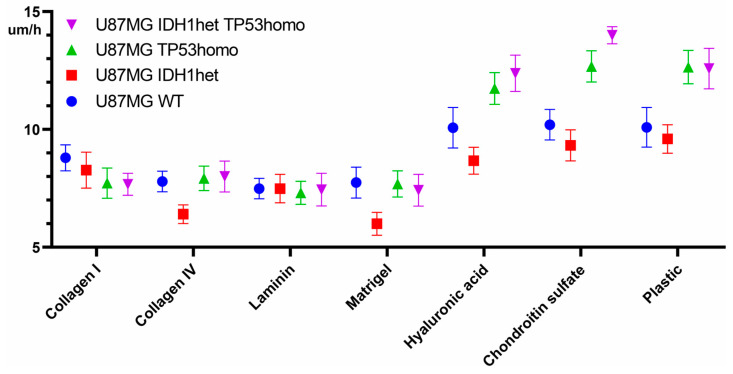
Comparison of cell migration with different mutational profiles, including combinations of mutations on different matrices.

The cell line harboring the genotype IDH1 R132H heterozygous (IDH1het) exhibits a significantly reduced migration rate on Matrigel and collagen IV compared to the other cell cultures (Figure 4). Specifically, the migration rates were recorded at 5.8 µm/h (95% CI of the median 5.1–6.4 µm/h) on Matrigel and 6.0 µm/h (95% CI of the median 5.7–6.1 µm/h) on collagen IV. In contrast, the parental U87MG cell line demonstrated median migration rates of 7.4 µm/h (95% CI of the median 6.6–8.4 µm/h) on Matrigel and 7.6 µm/h (95% CI of the median 7.0–8.4 µm/ho) on collagen IV. The U87MG TP53 R248Q heterozygous cells exhibited median migration rates of 7.4 µm/h (95% CI of the median 6.8–8.0 µm/h) on Matrigel and 7.8 µm/h (95% CI of the median 6.7–8.5 µm/h) on collagen IV.

Interestingly, the co-occurrence of TP53 R248Q homozygous with the IDH1 R132H heterozygous mutations restores the migratory capabilities (Figure 4). The median migration rates of U87MG IDH1 R132H heterozygous TP53 R248Q homozygous cells were estimated as 7.6 µm/h (95% CI of the median 6.5–8.3 µm/h) on Matrigel and 7.9 µm/h (95% CI of the median 7.3–8.6 µm/h) on collagen IV. No significant differences in migration rates were observed between the IDH1het-TP53homo cells and other cell cultures on collagen IV and Matrigel (*p* > 0.05). Furthermore, the IDH1 R132H mutation did not show significant effects on cell migration across collagen I and laminin (*p* > 0.05) (Figure 4).

### 2.5. Chondroitin Sulfate and Hyaluronic Acid Induce Cell Migration by Modulation of Cell Adhesion to the Surface

**Figure 5:**
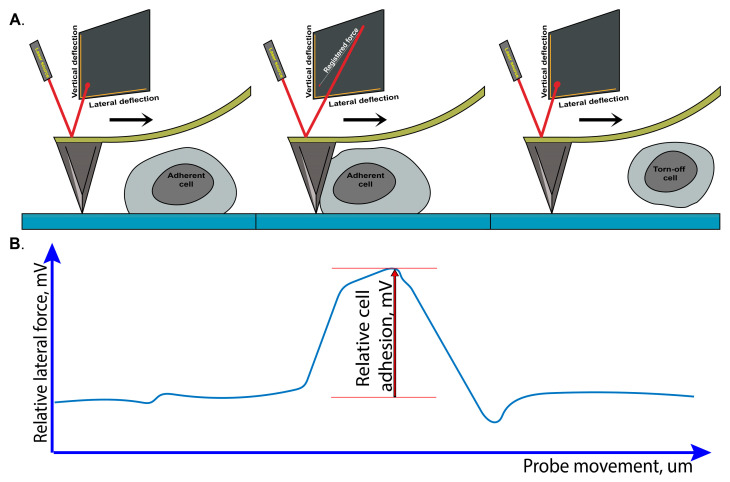
The procedure for cell adhesion assay based on atomic force microscopy: (**A**) Cantilever movement and probe–cell interaction. (**B**) Relative cell adhesion registration.

A protocol for measuring cell adhesion to the extracellular matrix using atomic force microscopy was optimized. The methodology involved two key steps: first, the lateral deflection of the cantilever was recorded, which was induced by the interaction between the probe and the cell (Figure 5A). Subsequently, the adhesion forces were determined by subtracting the background interaction from the peak probe–cell interaction force (Figure 5B).

**Figure 6:**
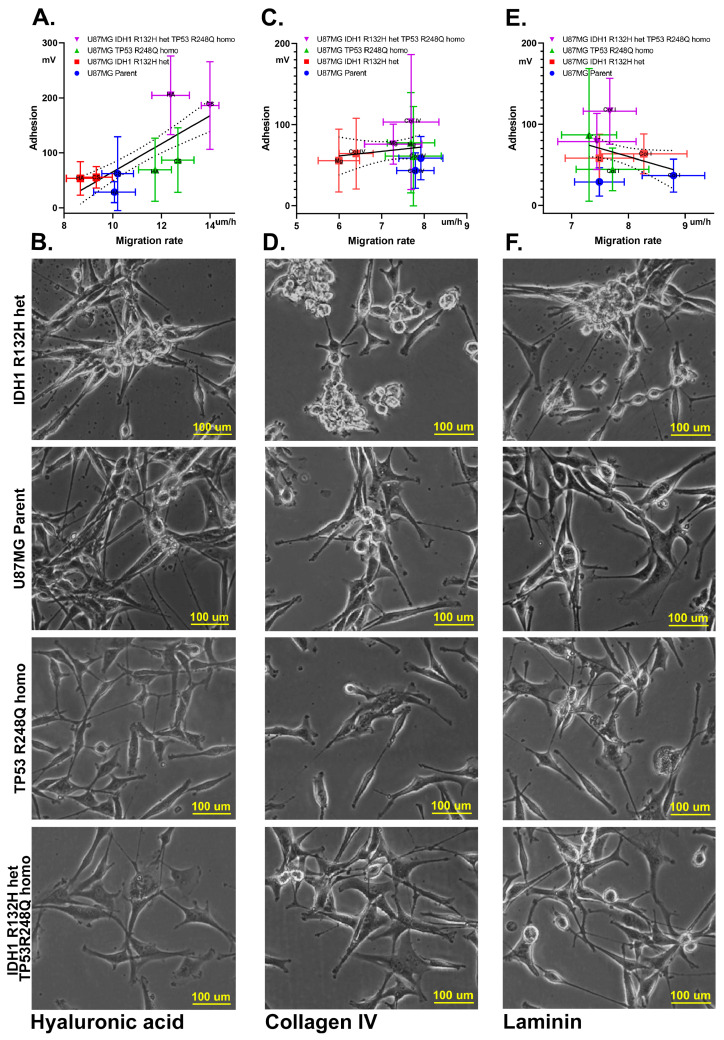
Correlation between cell adhesion and migration. (**A**) Scatterplot of cell adhesion and migration during cultivation on carbohydrate surfaces. (**B**) Typical cell morphology during cultivation on hyaluronic acid. (**C**) Scatterplot of cell adhesion and migration during cultivation on collagen IV and Matrigel. (**D**) Typical cell morphology during cultivation on collagen IV. (**E**) Scatterplot of cell adhesion and migration during cultivation on collagen I and laminin. (**F**) Typical cell morphology during cultivation on laminin.

All cell cultures exhibited elevated migration rates on hyaluronic acid and chondroitin sulfate. Notably, TP53homo and IDH1het-TP53homo cell lines showed significantly higher migration rates compared to those on protein-containing surfaces (*p* < 0.0002) (Figure 6A). Additionally, a strong Spearman correlation (r = 0.86, *p* = 0.01) was observed between the levels of cell migration and adhesion on the substrate.

Moreover, cells exhibiting the highest migration rates on polysaccharide surfaces also displayed increased surface areas and a more fibroblast-like morphology (Figure S1). The largest surface areas were identified in cell line IDH1het-Tp53homo (Figure 6B).

However, no significant correlation (*p* > 0.05) was found for all cell lines during cultivation on protein surfaces and Matrigel between cell adhesion and migration.

The cultivation of all cell cultures on Matrigel and collagen IV did not reveal significant differences in cell adhesion to the matrix or in migration patterns (Figure 6C). However, the IDH1het cell line displayed a distinctly altered cell morphology and spreading behavior by forming aggregates composed of round-shaped cells visually demonstrating decreased adhesion to the substrate (Figure 6D).

Conversely, when cultured on collagen I and laminin, all cell cultures exhibited comparable morphological characteristics, characterized by large amounts of convex spherical cells and a reduced number of flattened cells relative to those cultivated on other matrices (Figure 6F). Notably, the slope of the linear regression analysis indicated a trend towards negative values (Figure 6E).

## 3. Discussion

The effects of the IDH1 R132H and TP53 R248Q mutations remain poorly characterized, with unresolved questions and numerous gaps in our knowledge. Several studies have reported conflicting findings regarding cell migration (e.g., [19,21]), and there is a paucity of literature specifically addressing tumors harboring both IDH1 R132H and TP53 R248Q mutations. Furthermore, the ability of mutated cells to interact with ECM components has not been systematically investigated. This is particularly noteworthy given that the ECM of tumors undergoes significant alterations, characterized by the excessive production of specific protein and carbohydrate components, which, in turn, affects the overall rigidity and adhesion properties of the cells.

To investigate the impact of mechanical signaling on the invasion of cells exhibiting diverse molecular profiles, we estimated cell adhesion to various matrix components. We implemented a lateral shift adhesion assay to evaluate cell adherence by measuring the probe–surface friction. We adapted the protocol established by Nguyen et al. (2016) to optimize the disaggregation of cells for passivation on the matrix and the incubation duration necessary for accurate adhesion assessment, while minimizing the influence of endogenous matrix production [26]. However, to date, there has been no comparative study of the TP53 R248Q gain-of-function hotspot mutation in gliomas to determine the impact of these changes. A statistically significant correlation between the mutational profiles of cell cultures and their adhesion to components of the brain extracellular matrix and Matrigel was found. All studied cultures revealed a substantial role for chondroitin sulfate and hyaluronic acid in facilitating cell migration due to matrix adherence being a key factor in glioma cell migration [27]. Specifically, high concentrations of chondroitin sulfate and hyaluronic acid appear to decrease the synthesis of polysaccharide receptors, thereby supporting the hypothesis that these polysaccharides function as primary drivers of migration in glioma cells [28].

The elevated migration rate observed in the TP53 mutant cell line on carbohydrate surfaces can be attributed to increased expression levels of the HMMR receptor, which is responsible for the molecular recognition of HA and CS. The double-mutant genotype produced by consequently introducing IDH1 R132H and TP53 R248Q to parental glioblastoma cells was characterized by the highest migration among all the cell lines, with particular faster motility on chondroitin sulfate. Furthermore, the IDH1het-TP53homo cell line demonstrates elevated CD44 expression linked to the presence of the IDH1 R132H mutation. In contrast, the high expression of HMMR in this cell line is associated with the presence of the TP53 R248Q mutation. The highest migration capacity of the double-mutant cell line is associated with a synergic effect of the high expression of CD44 and HMMR, both responsible for an interaction with ECM glycans, specifically HA and CS [29]. CS is often considered not as a free component of the brain matrix but as a component of ECM proteoglycans and membrane-associated highly glycosylated molecules. However, chondroitin sulfate is an important factor in cell proliferation [30]. Thus, versican is a large membrane chondroitin sulfate-containing proteoglycan known for its role in cell migration and interactions, and it has been proposed as a target for glioma therapy [31]. HA, known for its role in promoting cell proliferation and migration, appears to enhance the migratory capacity of cells, likely due to its ability to interact with cell surface receptors such as CD44 and annexins [30]. This interaction not only facilitates adhesion but also activates signaling pathways that promote cytoskeletal rearrangement and motility [32]. It has been shown that, in both three-dimensional and two-dimensional matrices, hyaluronic acid acts as the most important component, determining the activity of cell migration through the interaction of HA-CD44-ezrin [33,34]. Ezrin is a peripheral cytoplasmic protein that binds the membrane receptor and actin, which may indicate a mechanosensory nature of this mechanism [35].

Introduction of the IDH1 R132H mutation into glioma cells resulted in a significant reduction in the migratory ability on collagen IV, an important component of the brain ECM. At the same time, the co-occurrence of the TP53 R248Q with the IDH1 R132H mutation leads to a recovery high migratory ability on collagen IV. IDH1 R132H heterozygous cells are known for their diminished proliferation in vitro and in vivo, attributed to downregulation of the YAP-TAZ signaling pathway and mechanosensing capabilities partially due to the aberration of CDH2- and ITGAV-dependent pathways [36,37]. Our findings corroborate this, as the IDH1 R132H cells showed significantly lower adhesion to various matrices. Notably, a 2-hydroxyglutarate-dependent mechanism may further enhance the migratory capabilities of IDH1 mutant cells [38]. These findings suggest that the co-occurrence of TP53 R248Q homozygous plays a crucial role in modulating these effects. Apparently, the existing differences in the effect of the IDH1 R132H mutation on the migratory activity of glioma cells are due to the mechanism of mutation occurrence and the cell microenvironment. The migratory activity of glioma cells in vivo with the IDH1 R132H/wt genotype is regulated by high levels of HA and CS in the tumor, while cells with low migratory potential are apparently selected [39,40]. A high malignancy of gliomas with the IDH1 R132H and TP53 R248Q mutations is due to both substitutions, which is also determined by a high level of CS synthesis in gliomas, especially in high-grade astrocytoma and glioblastoma [41].

The differential effects of IDH1 R132H and TP53 R248Q mutations on cell migration highlight the complexity of cancer biology, where genetic alterations can significantly influence cellular interactions with the extracellular matrix. The enhanced migration associated with the TP53 R248Q mutation, coupled with the reduced migration observed in IDH1 R132H mutant cells, highlights the need to further explore how specific genetic patterns and their combinations may determine cell behavior in the tumor microenvironment. These findings may have important implications for therapeutic strategies aimed at targeting cell migration and invasion in cancer treatment. The high invasion rate of TP53-mutated gliomas is likely provided by high sensitivity of the cells to the HA and CS, combined to the hyperproduction of these polysaccharides in brain tumors [39,42].

## 4. Materials and Methods

### 4.1. Cell Lines

The human glioblastoma U87MG cell line (ATCC, Manassas, VA, USA) and its genetically modified derivatives were used for the investigation of cell migration and adhesion on various ECM components. U87MG is one of the most widely used glioblastoma cell lines in research. Its extensive characterization in the literature provides a solid foundation for comparison with other studies. U87MG cells exhibit characteristics that are representative of glioblastoma, including aggressive growth and invasive properties. This makes them a relevant model for studying the molecular mechanisms underlying glioma progression and metastasis. The U87MG cell line is characterized with chromosomal stability and proliferative activity compared to other lines (U251MG, T98G and U118MG), which is essential for the genetic engineering experiments. Using CRISPR-Cas9 protocol with cytidine base editors, the most important polymorphisms in the IDH1 and TP53 genes typically occurring in glioma subtypes were introduced to U87MG glioma cells initially characterized by the wild-type genetic variants. The base editor plasmids pBT277 and pBT375 were employed to induce TP53 R248Q polymorphism, while pEF-BFP and pEF-AncBE4max were utilized to induce the IDH1 R132H variant. Guide RNAs were generated using an online tool [43].

Following the editing procedure, several cell lines were established: U87MG-IDH1het, which harbors the heterozygous R132H mutation in the IDH1 gene; U87MG-TP53homo, carrying the homozygous R248Q mutation in the TP53 gene; and U87MG-IDH1het-TP53homo, which contains both the heterozygous R132H mutation in the IDH1 gene and the homozygous R248Q mutation in the TP53 gene. All cell lines were cultured in DMEM supplemented with L-Glutamine (Capricorn Scientific, Tucson, AZ, GmbH), along with 10% fetal bovine serum (FBS, Capricorn, Ebsdorfergrund, Germany), 100 U/mL penicillin and 100 μg/mL streptomycin. Cultures were maintained at 37 °C in a humidified atmosphere containing 5% CO_2_. Upon reaching approximately 70% confluence, the cells were harvested, washed with phosphate-buffered saline (PBS), resuspended and prepared for experimental use.

To verify the genotype stability and confirm the presence of the induced mutations, all cell lines underwent Sanger sequencing. Additionally, prior to each experiment, cell cultures were screened for mycoplasma contamination using quantitative polymerase chain reaction (qPCR).

### 4.2. Cell Genotyping and Sanger Sequencing

Genomic DNA was extracted from 0.5 million U87MG cells utilizing the GeneJET genomic DNA purification kit (Thermo Scientific, Waltham, MA, USA) in accordance with the manufacturer’s instructions. The cell lines were screened through PCR amplification of the target DNA fragment derived from the cell lysate. The lysate was subsequently combined with the PCR mix, AccuStart II PCR SuperMix (VWR, Radnor, PA, USA), along with specific primers designed for the amplification of IDH1 or TP53 gene fragments (Table S1). The resulting amplified PCR products were purified using a PCR purification kit (Qiagen, Hilden, Germany) and prepared for sequencing.

For the screening of the cell lines, gene fragments encompassing the regions of interest for IDH1 and TP53 were amplified using DreamTaq Green PCR Master Mix (2×) (Table S2) (Thermo Scientific, Waltham, MA USA). A total of 10 ng of purified DNA was utilized for the Sanger sequencing reaction, employing the BigDye™ Terminator v3.1 cycle sequencing kit (Applied Biosystems, Foster City, CA, USA). The sequencing reaction was subsequently purified using the BigDye XTerminator™ Purification Kit (Applied Biosystems, Waltham, MA, USA) and analyzed on a 3500 Genetic Analyzer (Applied Biosystems, Waltham, MA, USA). Chromatogram analysis was conducted using SnapGene Viewer software (GSL Biotech, San Diego, CA, USA).

### 4.3. Preparation of Coatings

Six different coating materials were used to coat culture plastics prior to cell cultivation. The collagen I type extracted from rat tail tendons [44] was diluted in 30 mM acetic acid; a mix of laminins isolated from donor placenta [45] was diluted in 2.0 M Urea, 0.5 M NaCl, 0.02 M Tris-HCl, pH 7.4 and collagen IV type purified from chicken gizzard [46]. Matrigel (Corning, Corning, NY, USA), sodium hyaluronate and chondroitin 6-sulfate (Sigma-Aldrich, St. Louis, MO, USA) were prepared in PBS. All solutions of the ECM components had final concentrations of 10 µg/mL and were incubated 1 h at 37 °C for coating [47,48,49,50,51,52,53]. Hence, excess solutions were ejected by pipette. Coatings of laminin, collagens I and IV types, Matrigel and polystyrene were washed by PBS. Simultaneously, coatings of sodium hyaluronate and chondroitin 6-sulfate were completely dried at 60 °C [50,51,52]. All substrates were rinsed with PBS before cell cultivation.

### 4.4. Cell Migration Assay

The migratory capacity of the cells was assessed using high-content microscopy on the Cell IQ system (CM Technologies, Tampere, Finland), which employs vital imaging and machine learning algorithms for cell recognition. A total of 10 000 cells/cm² were seeded onto precoated polystyrene 24-well plates (Greiner Bio-One, Frickenhausen, Germany). Cells were incubated for a minimum of 30 min under standard culture conditions prior to the commencement of the experiment to allow the cells to sink to the bottom of the coated plate for better Cell IQ focusing.

The migration capacity was evaluated over a period of five days using a dry microscope objective (Olympus UPLFLN 10×, Tokyo, Japan) under standard culture conditions. Nine time lapse fields of view were captured for each well.

The Cell IQ CellFinder algorithms for cell shape recognition were developed by selecting cells from the acquired image series. Subsequently, the images were analyzed using the established algorithms to identify all cells, allowing for the calculation of cell displacement relative to their previous positions. Migration tracks and average speed of movement (μm/h) were computed based on the cell offset data.

### 4.5. Immunocytochemical Assay

Cells were fixed using a 2% paraformaldehyde solution prepared in phosphate-buffered saline (PBS) for 15 min. Following fixation, cells were washed three times for 5 min each in 0.05% Tween-20 (Helicon, Moscow, Russia) diluted in PBS (PBS-T).

Membrane permeabilization was achieved by incubating the cells in a 0.5% Triton X-100 (Helicon, Moscow, Russia) solution prepared in PBS for 5 min at room temperature. Subsequently, cells were washed three times for 5 min each in PBS-T to eliminate residual Triton X-100. To minimize non-specific antibody binding, cells were incubated for 2 h with 3% bovine serum albumin (Sigma-Aldrich, Burlington, MA, USA) prepared in PBS. Prior to the addition of the antibody solution, cells were rinsed three times for 5 min each in PBS-T. Cell labeling was performed using primary antibodies against CD44 (ab157107, Abcam, Cambridge, UK) at the recommended dilution in PBS for 2 h at room temperature. To further reduce non-specific binding, cells were rinsed three times for 5 min each in PBS-T prior to the application of secondary antibodies. Secondary antibody labeling was conducted using Alexa Fluor 488 goat anti-rabbit IgG (H + L) (a11034, Thermo Fisher, Waltham, MA, USA) and Alexa Fluor 546 goat anti-mouse IgG (H + L) (a11003, Thermo Fisher, Waltham, MA, USA) for 1 h at room temperature. Following this incubation, cells were subjected to three additional rinses in PBS-T for 5 min each to minimize non-specific fluorescence.

Nuclear staining was performed by adding DAPI (Sigma-Aldrich, USA) at a final concentration of 300 nM for 10 min during the final washing step.

### 4.6. Atomic Force Microscopy

Atomic force microscopy (AFM) experiments were conducted under controlled environmental conditions at 25 °C. Sharp SNL-A probes (Bruker, Billerica, MA, USA) were utilized, and prior to each session, the probes were calibrated to determine the deflection sensitivity, spring constant and tip radius. For the analysis, we employed NanoScope analysis software (Bruker, Billerica, MA, USA), which was provided alongside the AFM system.

### 4.7. Cell Adhesion Assay

To assess the degree of adhesive capacity of cells with different genetic profiles, a method for measuring cell adhesion based on contact atomic force microscopy with the registration of lateral cantilever deflection–lateral shift assay (AFM-LSA) was used. A RTESPA-150 (Bruker, Billerica, MA, USA) probe was applied for the assay. During the experiment, cell cultures were seeded on dishes coated with extracellular matrix components: hyaluronic acid, chondroitin sulfate, type I collagen, Matrigel, laminin and type IV collagen. In total, the adhesion degree of 4 cell lines was assessed: U87MG, U87MG-IDH1het, U87MG-TP53homo and U87MG-IDH1het-TP53homo. Cell cultures were incubated on the scaffolds for 25 min and then quickly fixed with 70% ethyl alcohol to offset the effect of differences in the adhesion time.

### 4.8. qPCR Gene Expression Assay

Gene expression analysis was performed by qPCR. Total RNA was extracted from 1–1.5 × 10^6^ cells using a Lira+ LRP-100–3 kit (Biolabmix, Novosibirsk, Russia). The RNA concentration was measured by NanoPhotometer P330 (Implen, San Diego, CA, USA), and the integrity was confirmed by the A260/280 ratio and 1.5% agarose gel electrophoresis. We obtained RNA concentrations of 225 ng/µL, and A260/280 was 1.7–2.0. Then, 1 µg of total RNA was reverse transcribed using the MMLV RT kit (Evrogen, Moscow, Russia) according to the manufacturer’s guidelines.

The qPCR was performed using HS-qPCR SYBR Blue (2×) MHC030–2040 (Biolabmix, Novosibirsk, Russia). Before gene expression analysis, the PCR condition for all primer pairs was optimized using 10-fold cDNA serial dilution to gain an efficiency of 90–110% (primers sequences, annealing temperatures and final primers concentrations see in Table S3). The reaction ran in Real-Time CFX96 Touch (Bio-Rad, Hercules, CA, USA) with the following protocol: 95 °C for 5 min for initial denaturation and 45 cycles of denaturation at 95 °C for 10 s, annealing at 60 °C for 15 s, extension 72 °C for 20 s and melt curve analysis at 65–95 °C by 0–5 °C at 3 s/step. The results were analyzed with CFX Manager software (Bio-Rad, Hercules, CA, USA). The mRNA expression levels were normalized to the internal standard CSNK2B [54]. Relative gene expression was measured according to the double delta Ct (ddCt) analysis.

### 4.9. Statistical Analysis

Statistical analysis was conducted using GraphPad Prism software ver. 8.0.1. (GraphPad Software Inc., San Diego, CA, USA). Non-parametric statistics were employed, specifically the Kruskal–Wallis test, followed by Dunn’s multiple comparison test, to evaluate differences among multiple datasets. Additionally, Spearman’s correlation was utilized to assess the dependencies between variables.

## Data Availability

Data are contained within the article and supplementary materials.

## Supplementary Materials

The following supporting information can be downloaded at https://www.mdpi.com/article/10.3390/ijms252212178/s1: Figure S1. Representative images on all cell cultures cultivated on all matrices. Table S1. gRNA sequences for targeting IDH1 and TP53 genes. Table S2. Primer sequences to check for glioma mutations. Table S3. Primers for qPCR assay
