# Supplementary material for: IDH1 R132H and TP53 R248Q Mutations Modulate Glioma Cell Migration and Adhesion on Different ECM Components
Source: Int J Mol Sci. 2024 Nov 13;25(22):12178. doi: 10.3390/ijms252212178 (PMC11594609; doi:10.3390/ijms252212178)
Supplement: Supplementary file 1 [file ijms-25-12178-s001.zip › Tables S1 S2 S3.pdf]

International Journal of
Molecular Sciences

MDPI

Table S1. gRNA sequences for targeting IDH1 and TP53 genes.

The primer title	5′-3′ sequence
TP53_R248Q_AGG_VG_F	CACCgcggttcatgccgcccatgc
TP53_R248Q_AGG_VG_R	AAACgcatgggcggcatgaaccgc
TP53_R248Q_TGC_VG_F	CACCgctccggttcatgccgccca
TP53_R248Q_TGC_VG_R	AAACtgggcggcatgaaccggagc
IDH1_R132H_Cas9_Be3_Fwd	CACCgcatgacgacctatgatgat
IDH1_R132H_Cas9_Be3_Rev	AAACatcatcataggtcgtcatgc

Table S2. Primer sequences to check for glioma mutations.

Primer name	5′-3′-sequence
IDH1_PCR_Fwd	GTTGAAACAAATGTGGAAATCACC
IDH1_PCR_Rev	TTCATACCTTGCTTAATGGGTGT
IDH1_Seq*	GTTGAAACAAATGTGGAAATCACC
IDH2_PCR_Fwd	GCTGCAGTGGGACCACTATTA
IDH2_PCR_Rev	CAGAGACAAGAGGATGGCTAGG
IDH2_Seq*	CTGTCCTCACAGAGTTCAAGC
TP53_R175_F	TTTTGCCAACTGGCCAAGACCT
TP53_R175_R	GCCAGACCTAAGAGCAATCAGTG
TP53_R248_F	GAAACCCCGTCTCTACTGAA
TP53_R248_R	GAAGAAATCGGTAAGAGGTGGGC
TP53_R273_F	GGGAGTAGATGGAGCCTGGTTTTTT
TP53_R273_R	GCTTCTTGTCCTGCTTGCTTACC

Table S3. Primers for qPCR assay

<table>
<tr><th>Primer</th><th>Sequence</th><th>Final concentration in reaction</th><th>Annealing temperature (0C)</th></tr>
<tr><td>CSNK2B_qPCR_F</td><td>TGGAGCCTGATGAAGAACT</td><td>400nM</td><td rowspan="2">60</td></tr>
<tr><td>CSNK2B_qPCR_R</td><td>GGTTGGTAAGGATGTAGCG</td><td>400nM</td></tr>
<tr><td>CD44_qPCR_F</td><td>TGGACAAGTTTTGGTGGC</td><td>500nM</td><td rowspan="2">60</td></tr>
<tr><td>CD44_qPCR_R</td><td>CCGAGAGATGCTGTAGCG</td><td>500nM</td></tr>
</table>

CDH2_qPCR_F	TCCAGAGTTTACTGCCATGAC	500nM	60
CDH2_qPCR_R	GGATCTCCGCCACTGATTC	500nM	
HMMR_qPCR_F	AGCAAACACTGGATGAGC	250nM	60
HMMR_qPCR_R	CCTGGGTATGAGCAGCAC	250nM	
ITGV_qPCR_F	TGACTGGTCTTCTACCCG	500nM	60
ITGV_qPCR_R	TCTTGTGATCTACATGGAGC	500nM	
VIM_qPCR_F	GCAAAGCAGGAGTCCACTGAG	500nM	60
VIM_qPCR_R	CTTAACATTGAGCAGGTCTTGGTAT	500nM	
